# Ferroelectricity by Bose–Einstein condensation in a quantum magnet

**DOI:** 10.1038/ncomms12822

**Published:** 2016-09-26

**Authors:** S. Kimura, K. Kakihata, Y. Sawada, K. Watanabe, M. Matsumoto, M. Hagiwara, H. Tanaka

**Affiliations:** 1 Institute for Materials Research, Tohoku University, Sendai 980-8577, Japan; 2 Department of Physics, Shizuoka University, Shizuoka 422-8529, Japan; 3 Center for Advanced High Magnetic Field Science, Graduate School of Science, Osaka University, Toyonaka 560-0043, Japan; 4 Department of Physics, Tokyo Institute of Technology, Tokyo 152-8551, Japan

## Abstract

The Bose–Einstein condensation is a fascinating phenomenon, which results from quantum statistics for identical particles with an integer spin. Surprising properties, such as superfluidity, vortex quantization or Josephson effect, appear owing to the macroscopic quantum coherence, which spontaneously develops in Bose–Einstein condensates. Realization of Bose–Einstein condensation is not restricted in fluids like liquid helium, a superconducting phase of paired electrons in a metal and laser-cooled dilute alkali atoms. Bosonic quasi-particles like exciton-polariton and magnon in solids-state systems can also undergo Bose–Einstein condensation in certain conditions. Here, we report that the quantum coherence in Bose–Einstein condensate of the magnon quasi particles yields spontaneous electric polarization in the quantum magnet TlCuCl_3_, leading to remarkable magnetoelectric effect. Very soft ferroelectricity is realized as a consequence of the *O*(2) symmetry breaking by magnon Bose–Einstein condensation. The finding of this ferroelectricity will open a new window to explore multi-functionality of quantum magnets.

## Abstract (web summary)

Magnons, quantized spin excitations in magnetic materials, may undergo Bose-Einstein condensation into a macroscopic correlated quantum state at low temperature. Here, the authors demonstrate how magnon condensation in quantum magnet TlCuCl_3_ generates an electrical polarization.

## Graphical abstract

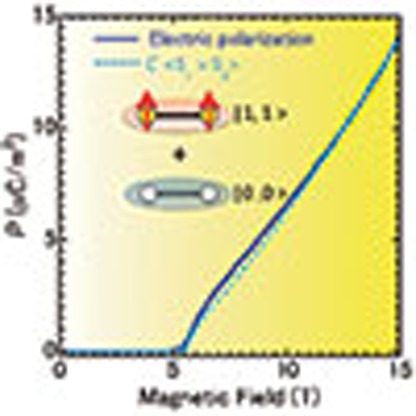

TlCuCl_3_ is the first material whose field-induced quantum phase transition was classified as a realization of Bose–Einstein condensation (BEC) in the quantum magnets^1^. In this material, antiferromagnetic spin dimers, composed of a pair of Cu^2+^ ions with spin *S*=1/2, are three-dimensionally coupled by interdimer Heisenberg exchange interactions, which are weaker than the intradimer one^2^,^3^,^4^,^5^. This coupled dimer lattice provides an ideal playground for BEC of magnon quasiparticles. The ground state of the system is approximated to be direct product of the spin singlet states on the dimer bonds. Thus, TlCuCl_3_ remains to be a quantum paramagnet down to the lowest temperature, while the excited triplet on a dimer, propagating through the lattice via the interdimer exchange interactions, can be regarded as a Bosonic particle with *S*=1, so called magnon. Owing to the interdimer exchange interactions, the magnon becomes dispersive. The magnon dispersion has a minimum of its excitation energy at wave vector **Q**=(0, 0, 2π) with a finite gap Δ/*k*_B_∼7.5 K from the ground state^4^,^5^. The external magnetic field causes a splitting of the triply degenerate dispersion of magnon into three branches with magnetic quantum number *S*_*Z*_=+1, 0, −1 (ref. 6), where the *z*-axis is defined to be the direction of the external magnetic field. Therefore, the magnon branch with *S*_*Z*_=+1 becomes soft in magnetic fields, and then closing of the energy gap occurs at a critical field *H*_c_=Δ/*gμ*_B_∼5.5 T (refs 6, 7). Once the gap is closed, BEC of the magnon takes place, leading to the field-induced long range ordering of the transvers spin components^1^,^2^,^3^,^8^,^9^,^10^,^11^,^12^,^13^. An important feature, which we notice for our finding in the present work, is that the condensate above *H*_c_ has a finite expectation value of a quantum-mechanical operator **S**_*i*_ × **S**_*j*_, which is an outer product of neighbouring spins. This quantity, namely the vector spin chirality, is a key ingredient for the colossal magnetoelectric coupling, which was found in helical magnetically ordered materials.

The magnetoelectric coupling offers efficient control of the electric polarization by magnetic fields and the magnetization by electric fields, and might achieve a breakthrough in development of new multifunctional devices. Studies for the magnetoelectric coupling have become much more intensive, since the magnetoferrolectricity, in which the spontaneous electric polarization is induced by helical magnetic ordering, was discovered in manganese perovskite oxides^14^. Subsequent theoretical studies clarified a close connection between the electric polarization and the vector spin chirality. The microscopic calculation by Katsura, Nagaosa and Balatzky derived a spin-dependent electric dipole moment **p**∝**e**_*i,j*_ × (**S**_*i*_ × **S**_*j*_), which appears under the influence of spin orbit interaction^15^. Here, **e**_**i,j**_ is a vector connecting between the two spin sites. This calculation indicated that the vector spin chirality could be a source of the electric polarization^15^,^16^. The helical magnetic order can be regarded as ferroic order of the vector spin chirality, and therefore results in the appearance of the spontaneous electric polarization^16^. Significantly, the quantum spin dimer has a finite matrix element of this vector spin chirality between its spin singlet and triplet states. This property leads to the emergence of the ferroelectricity by the magnon BEC in the coupled dimer system. In the quantum condensed state of the coupled dimer system, realized above *H*_c_, the wave function Ψ of an individual dimer is approximated by a coherent superposition of the singlet and the triplet with *S*_*Z*_=+1, given as 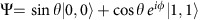
2,3,11. Here, *θ* is an angle, determined by a ratio between the exchange interactions and the Zeeman energy, whereas *φ* is an arbitrary phase, which reflects the rotational symmetry of the system around the external magnetic field. Because of the finite matrix element of **S**_1_ × **S**_2_ between the spin singlet and triplet states mentioned above, the wave function Ψ has a finite expectation value of this quantity as follows:


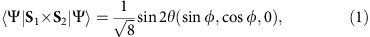


where **S**_1_ and **S**_2_ are the spins, composing a dimer. From this fact, we anticipate the appearance of the electric dipole moment in this superposed state. When the macroscopic coherence is developed throughout the dimer lattice by magnon BEC, the phase *φ* is settled and thereby the electric dipole moment with a fixed direction emerges on a dimer. Therefore, if a sum of this electric dipole on each dimer over whole lattice has a finite value, a macroscopic spontaneous electric polarization will appear in the system.

Here, we demonstrate ferroelectricity, caused by magnon BEC, by the dielectric constant and the pyroelectric current measurements of TlCuCl_3_. It is revealed that the spontaneous electric polarization proportional to an absolute value of the vector spin chirality in the ground state develops above *H*_c_. The observation of this ferroelectricity indicates that quantum magnets can be significant playgrounds for magnetoelectric coupling.

## Results

### Ferroelectricity in TlCuCl_3_

**Figure 1:**
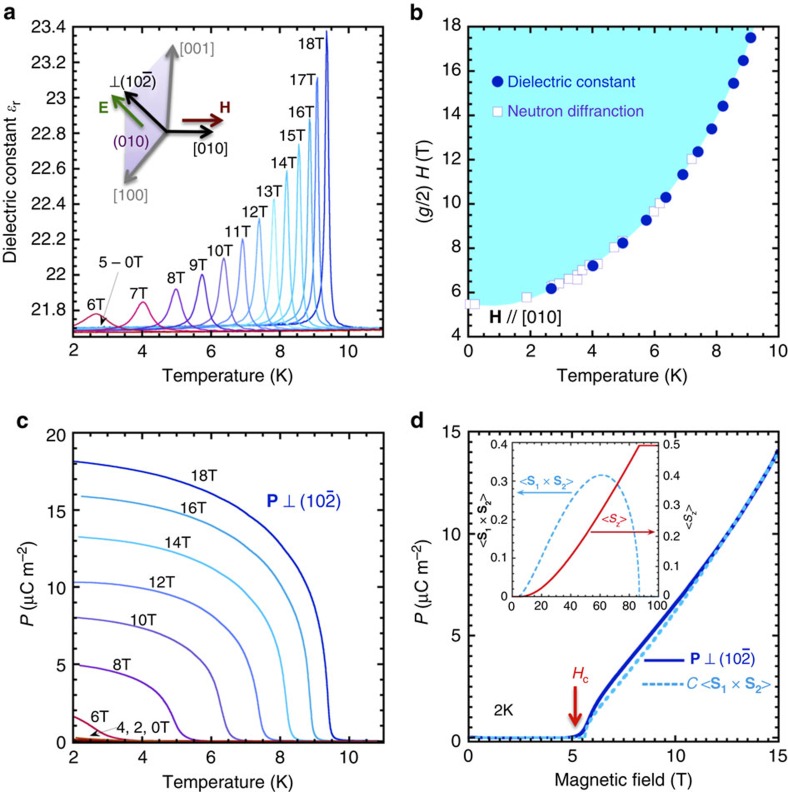
Ferroelectricity by magnon BEC in TlCuCl_3_. (**a**) Temperature dependence of the dielectric constant observed in AC electric fields **E** perpendicular to 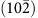 plane. The inset shows the experimental configuration of the measurement. (**b**) Temperature-field phase diagram. Closed circles shows the peak position of the dielectric constant. Open squares are the critical points, determined by the previous neutron diffraction measurements^13^. The light blue shedding area turns out to be a multiferroic state with both antiferromagnetic and ferroelectric orders. The magnetic field is normalized by the *g*-value *g*=2.06, evaluated by the ESR measurement^10^. (**c**) Temperature dependence of the electric polarization **P**⊥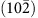. (**d**) Field dependence of the electric polarization **P**⊥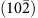. A solid curve shows the experimental result. A dashed curve is field dependence of the calculated vector spin chirality <**S**_**1**_ × **S**_**2**_>, multiplied by a constant *C*=220 (μC m^−2^). The calculation is carried out in terms of the bond operator theory. Inset shows the field dependence of the vector spin chirality <**S**_**1**_ × **S**_**2**_> and the longitudinal spin component <*S*_z_>, calculated up to 100 T.

To observe the ferroelectricity by magnon BEC, we have measured the dielectric constant (*ɛ*) and the spontaneous electric polarization (**P**) in TlCuCl_3_. Figure 1a shows the temperature dependence of the dielectric constant of TlCuCl_3_ perpendicular to 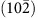 plane in magnetic fields (**H**) for **H**//[010]. While no anomaly is found in the dielectric constant observed below 5 T, a peak appears at low temperature in magnetic fields above 6 T. As the magnetic field is increased, the peak becomes sharper and shifts towards higher temperature. The positions of the observed peaks are plotted in the temperature-field phase diagram shown in Fig. 1b. The vertical field axis of Fig. 1b is normalized by the *g*-value *g*=2.06 determined by the electron spin resonance (ESR) measurement^10^. The peak positions agree with the critical points of the magnon BEC, determined by the previous neutron diffraction measurements in TlCuCl_3_ (ref. 13). This agreement indicates that the magnon BEC is accompanied by a sharp anomaly of the dielectric property, implying that the BEC phase in TlCuCl_3_ is a multiferroic state with both ferroelectic and antiferromagnetic ordering. The appearance of the spontaneous electric polarization by the magnon BEC can be confirmed by the pyroelectric current measurements. Figure 1c shows the temperature dependence of the spontaneous electric polarization perpendicular to the 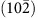 plane and in the magnetic field for **H**//[010], obtained by integrating the pyroelectric current. As expected, the electric polarization appears below the critical temperature in magnetic fields above 6 T. As the magnetic field is increased, the saturation value of the electric polarization at the lowest temperature increases.

### Magnetic field dependence of electric polarization in TlCuCl_3_

Figure 1d shows the field dependence of the electric polarization, observed at 2.0 K in the same configuration for the temperature dependence measurements described above. Continuous increase of the electric polarization with increasing the magnetic field is observed above *H*_*c*_. We compare the field evolution of the electric polarization to the expectation value of the vector spin chirality <**S**_1_ × **S**_2_> of a coupled dimer system in the condensed ground state, calculated in terms of a bond operator formulation. As described in refs 2, 3, the bond operator formulation can quantitatively explain the magnetic field dependence of both longitudinal and transverse spin components, observed in TlCuCl_3_, by using the exchange interaction parameters, determined from the analysis of the magnon dispersion at zero magnetic field. The dashed line in Fig. 1d shows the field dependence of *C* <**S**_1_ × **S**_2_> calculated with a constant *C*=220 [μC m^−2^] and the same exchange parameters listed in ref. 3. A good agreement between the experiment and the calculation, seen in Fig. 1d, indicates that the electric polarization in TlCuCl_3_ is caused by the emergence of the vector spin chirality in the condensate, developed above *H*_c_. Inset of Fig. 1d shows the field dependence of <**S**_1_ × **S**_2_> and the longitudinal spin component <*S*_*z*_>, calculated up to 100 T. The calculation suggests that the electric polarization in TlCuCl_3_ reaches a maximum around 60 T, and then disappears around 87 T, where the spins are completely polarized to the direction of the external magnetic field.

## Discussion

**Figure 2:**
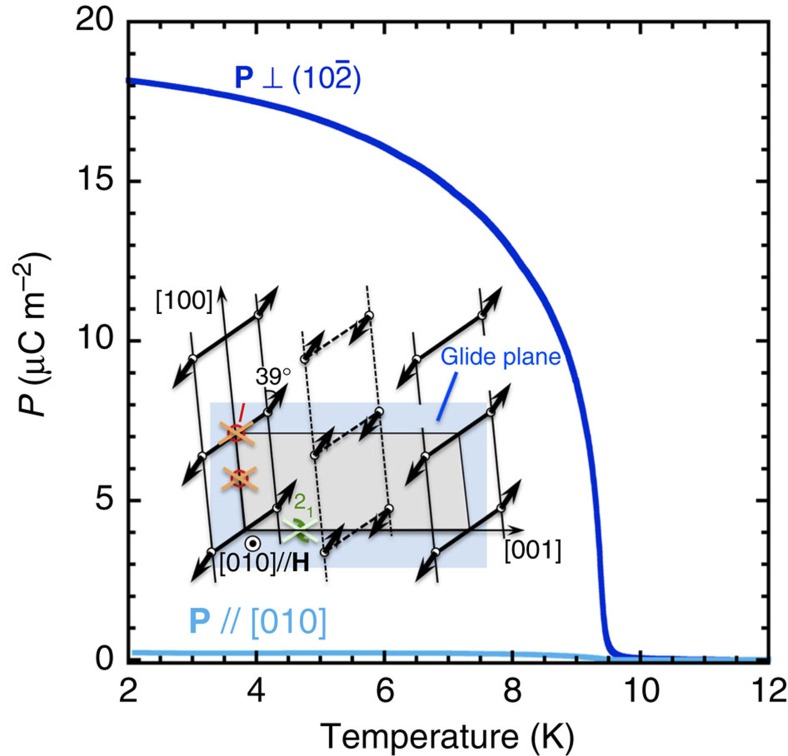
Temperature dependence of the electric polarizations P//[010] and P⊥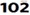 for H//[010] at 18 T. Inset shows the spin structure in the field-induced phase of TlCuCl_3_ for **H**//[010], determined by the previous neutron diffraction measurements^13^. Grey shedding area shows a unit cell of TlCuCl_3_. Bold solid and dashed lines show the dimer bonds, which are connected each other by the two-hold helical 2_1_ or the glide symmetry transformations of the *P*2_1_/*c*, which is the space group of TlCuCl_3_. Arrows are spins of Cu^2+^ ion. The transverse components of the spins direct along the easy axis, which is inclined from [100] by 39° in (010) plane^13^. The spin ordering breaks the space inversion *I* and the 2_1_ symmetries, whereas the glide plane parallel to (010) remains. Therefore, the electric polarization, induced by the magnetic field for **H**//[010], should lie in (010) plane. The experimental result that almost no **P**//[010] is observed, agrees with the symmetry of the spin structure.

Let us discuss the relation between the direction of the electric polarization and the symmetry of the field-induced BEC phase in TlCuCl_3_. Inset of Fig. 2 shows the spin structure of the BEC phase in the magnetic field parallel to [010] axis, determined by the previous neutron diffraction measurement^13^. The crystal symmetry of TlCuCl_3_ belongs to the *P*2_1_/*c* space group^17^. However, in magnetic fields for **H**//[010], the magnetic order, which is characterized by wave vector **Q**, breaks the space inversion and the twofold helical symmetries of the crystal lattice. Only a remaining element of the symmetry in the BEC phase is a glide plane parallel to (010). Therefore, the electric polarization, induced by external magnetic fields for **H**//[010], should lie in (010) plane. Figure 2 exhibits the temperature dependences of the electric polarizations perpendicular to 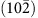 plane and parallel to [010] axis in magnetic fields for **H**//[010]. The experimental result that almost no electric polarization appears for [010] direction, indicates that the electric polarization points along (010) plane as expected from the symmetry of the BEC phase.

**Figure 3:**
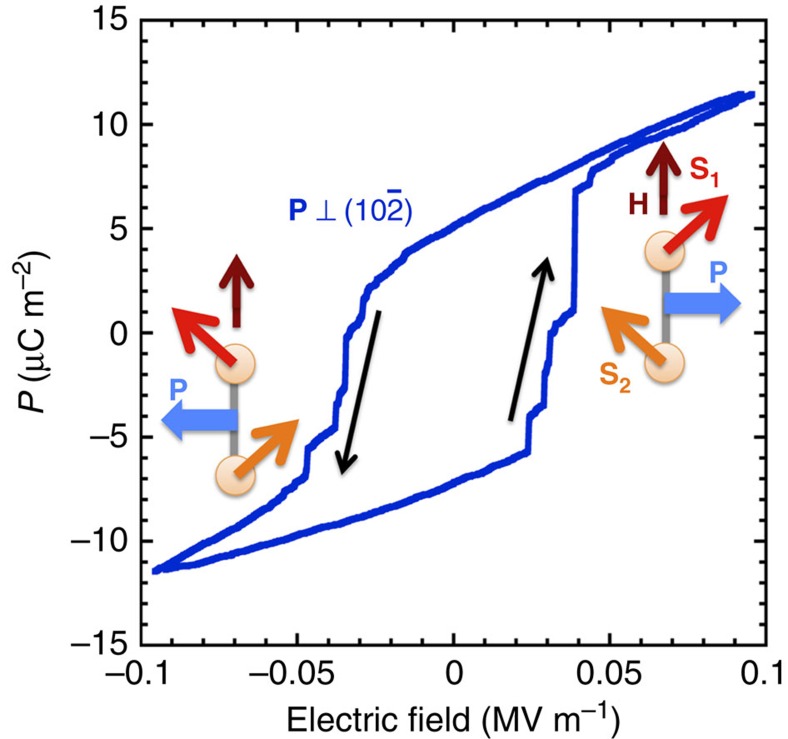
*P*-*E* hysteresis curve of P⊥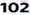 for H//[010]. Small electric coercive field for reversal of **P** indicates that 180° rotation of the antiferromagnetic domain easily occurs in TlCuCl_3_. The insets show schematic pictures of the electric polarization on the dimer. The electric polarization reversal requires a reversal of the vector spin chirality **S**_**1**_ × **S**_**2**_.

Figure 3 shows a *P*–*E* hysteresis loop at 4.2 K for the electric polarization perpendicular to 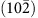 plane, observed for **H**//[010] at 14 T. Step-like reversals of the electric polarization are observed around the electric coercive field *E*_r_=0.03 MV m^−1^. To the knowledge of the authors, this value of the electric coercive field is the smallest among the magnetically induced ferroelectrics. The electric polarization reversal requires a reversal of the vector spin chirality. Thus, it causes 180° rotation of the antiferromagnetic domain of the ordered spin structure around the external magnetic field. The small electric coercive field indicates that the antiferromagnetic domain can easily rotate. Therefore, the very low electric coercive field is explained by the small magnetic anisotropy in TlCuCl_3_. The concept of BEC in the quantum magnets was obtained from mapping the spin system onto a Bose gas of the interacting magnon quasiparticles^1^,^11^,^12^. A *U*(1) symmetry breaking in the condensate of the Bose gas corresponds to the spontaneous breaking of the rotational *O*(2) symmetry of the spin system, which results in the magnetic ordering of the transverse spin components. The correspondence between the Bose gas and the spin system is fully valid only for an ideal system with a continuous uniaxial symmetry around the magnetic field. Nevertheless, for real materials, in which a symmetry breaking magnetic anisotropy inevitably exists, the concept of BEC can be applied, if the magnitude of such an anisotropy term is low enough compared with the ordering temperature^11^,^12^,^18^. The very soft nature of the ferroelectricity in TlCuCl_3_ confirms that this material is the most suitable material to realize BEC in quantum magnet. Finally, we should mention about a vortex, which is a topological defect within the Bose–Einstein condensate. The vortex in the magnon Bose–Einstein condensate can have an electric polarization parallel to the vorticity vector. This could imply that a local electric field would allow to control the vortices and might even affect the nature of the phase transition by the magnon BEC.

## Methods

### Measurements of dielectric constant

A single crystal TlCuCl_3_, which was grown by the Bridgman method, was cut into thin plates with the widest plane parallel to cleavage (010) and 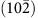 plane, and then silver paste were applied on faces of the crystal as electrodes. (010) and 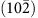 planes are orthogonal each other. We measured the dielectric constant at 10 kHz by an *LCR* metre (Agilent E4980A).

### Measurements of electric polarization

To obtain the temperature dependence of the spontaneous electric polarization, the pyroelectric current was measured after applying a poling electric field of 0.043 MV m^−1^ from 20 to 2 K by using an electrometer (Keithley 6517B). The magnetic field dependence of the electric polarization was obtained by integration of the electric current observed in sweeping magnetic field at a constant ratio 0.984 T min^−1^. The *P*–*E* curve was obtained by measurements of electric current induced by sweeping electric field at a constant ratio 21.75 kV m^−1^ s^−1^. Both dielectric and pyroelectric current measurements were carried out by utilizing a 18 T superconducting magnet.

### Data availability

The data that support the findings of this study are available from the corresponding author on request.

## Additional information

**How to cite this article:** Kimura, S. *et al.* Ferroelectricity by Bose-Einstein condensation in a quantum magnet. *Nat. Commun.* 7:12822 doi: 10.1038/ncomms12822 (2016).
